# Biological Characteristics, Domesticated Cultivation Protocol, Antioxidant Activity, and Protective Effects against Cellular Oxidative Stress of an Underutilized Medicinal Mushroom: *Fomitopsis palustris*

**DOI:** 10.3390/jof10060380

**Published:** 2024-05-25

**Authors:** Yi Liang, Dan Dai, Wan-Qiu Chang, Yang Wang, Zhen-Hao Zhang, Dan Li, Bo Zhang, Yu Li

**Affiliations:** 1 Engineering Research Center of Edible and Medicinal Fungi, Ministry of Education, Jilin Agricultural University, Changchun 130118, China; 2 Institute of Agricultural Applied Microbiology, Jiangxi Academy of Agricultural Sciences, Nanchang 330200, China; 3 College of Plant Protection, Shenyang Agricultural University, Shenyang 110866, China

**Keywords:** *Fomitopsis palustris*, brown-rot fungus, medicinal mushroom, orthogonal test, crude polysaccharide, antioxidant activity, RAW264.7cell

## Abstract

Brown-rot fungus is one of the important medicinal mushrooms, which include some species within the genus *Fomitopsis*. This study identified wild macrofungi collected from a broad-leaved tree in Liaoning Province as *Fomitopsis palustris* using both morphological and molecular methods. To elucidate the potential medicinal and economic value of *F. palustris*, we conducted single-factor and orthogonal tests to optimize its mycelium culture conditions. Subsequently, we completed liquid culture and domestic cultivation based on these findings. Furthermore, crude polysaccharides were extracted from the cultivated fruiting bodies of *F. palustris* and their antioxidant activity was evaluated using chemical methods and cell-based models. The results showed that the optimal culture conditions for *F. palustris* mycelium were glucose as the carbon source, yeast extract powder as the nitrogen source, pH 6.0, and a temperature of 35 °C. Moreover, temperature was found to have the most significant impact on mycelial growth. The liquid strains were fermented for 6 days and then inoculated into a cultivation substrate composed of broadleaf sawdust, resulting in mature fruiting bodies in approximately 60 days. The crude polysaccharides extracted from the cultivated fruiting bodies of *F. palustris* (FPPs) possess in vitro scavenging abilities against DPPH radicals and OH radicals, as well as a certain ferric-reducing antioxidant power. Additionally, FPPs effectively mitigated H_2_O_2_-induced oxidative stress in RAW264.7cells by enhancing the intracellular activity of antioxidant enzymes such as SOD and CAT, scavenging excess ROS, and reducing MDA levels. This study provides preliminarily evidence of the potential medicinal and economic value of *F. palustris* and offers initial data for the future development and utilization of this species.

## 1. Introduction

Brown-rot fungi degrade cellulose and hemicellulose in wood, causing brown rot in trees or wood. They are not only an important group of plant pathogens, but are also essential for maintaining forest nutrient cycles and ecosystem stability [1]. Notably, the vast majority of brown-rot fungi taxa are clustered in the order Polyporales, which is considered to be one of the important medicinal fungal resources. However, although some brown-rot fungi are of significant economic value as medicinal mushrooms, only a limited number of species are currently suitable for industrial cultivation using tree wood chips [1,2]. *Fomitopsis palustris* (Berk. & M.A. Curtis) Gilb. & Ryvarden (1985) belongs to the Basidiomycota, Agaricomycetes, Polyporales, and Fomitopsidaceae, and is typically found growing on living trees or fallen logs. The fruiting bodies are sessile and suberinite, initially juicy when fresh and harden upon drying, causing brown rot in wood. The species is widely distributed in regions such as Guangdong, Hainan, and Jilin in China, and has also been reported in countries such as the United States, India, and Japan. While there has been extensive research on the medicinal properties of certain species within the genus *Fomitopsis* P. Karst, such as *F. officinalis* and *F. pinicola* [3,4]. On the other hand, *F. palustris* has been more frequently studied for its enzyme activity, particularly in research on brown rot in wood [5,6,7]. Recent studies have revealed that an aqueous extract of its fruiting bodies and some triterpenoid derivatives exhibit cytotoxic effects against certain tumor cell lines [8], indicating that this species has potential in medicinal research in addition to its enzyme activity.

Free radical metabolism occurs in all tissues of the body. Reactive oxygen species (ROS), also known as reactive oxygen intermediates (ROIs), are by-products of normal cellular metabolism. Under normal circumstances, the body maintains a balance in the production and clearance of ROS. An appropriate amount of ROS has beneficial effects on some physiological processes, such as killing invading pathogens, promoting wound healing, and facilitating tissue repair. However, when the body is exposed to adverse stimuli, the production and clearance of ROS become unbalanced, and excessive ROS can cause serious problems to the body’s homeostasis. Much like the indiscriminate oxidation of exposed iron leads to rust, the ROS-dependent oxidation of intracellular biomolecules or organelles is thought to potentially lead to a range of diseases, including aging, cancer, and diseases of virtually all organs, among others [9,10,11,12]. Multiple studies have demonstrated that certain mushrooms exhibit remarkable functional effects, encompassing antioxidant, immunomodulatory, and anti-tumor activities. In particular, polysaccharides in mushrooms have attracted much attention because of their high efficiency, low toxicity, and wide availability [13,14,15,16,17,18,19].

This study identified macrofungi collected from a broad-leaved tree in Dalian City, Liaoning Province, using both morphology and molecular methods, as *F. palustris*. Currently, there are limited studies on the biological characteristics and antioxidant activity of *F. palustris*. To demonstrate the potential medicinal and economic value of this species, we determined the optimal cultivation conditions for its mycelium through single-factor and orthogonal tests, and completed liquid cultivation and domestication cultivation based on these findings. Subsequently, crude polysaccharides were extracted from cultivated fruiting bodies, and their antioxidant activities were evaluated using chemical methods and cell model methods. It is hoped that this study will provide some fundamental information for the future development and utilization of this species.

## 2. Materials and Methods

### 2.1. Materials and Chemicals

A new kit for extracting genomic DNA from plants was obtained from Cowin (Taizhou, China). 1,1-Diphenyl-2-picrylhydrazyl radical (DPPH) and vitamin C (VC) were obtained from Yuanye Bio-Technology Co., Ltd. (Shanghai, China). A Hydroxyl Radical Kit was obtained from Comin (Suzhou, China). A FRAP kit was obtained from Michy (Suzhou, China). RAW 264.7cells were purchased from the Cell Bank of the Chinese Academy of Sciences (Shanghai, China). Superoxide dismutase (SOD), catalase (CAT), and malondialdehyde (MDA) assay kits, and a total protein assay kit (with standard, BCA method) were obtained from the Institute of Biological Engineering of Nanjing Jiancheng (Nanjing, China). An ROS assay kit and CCK-8 assay kit were obtained from Beyotime Biotechnology (Shanghai, China). Other reagents and chemicals purchased from locally approved vendors were analytically pure.

### 2.2. Collection and Identification of Fomitopsis palustris

#### 2.2.1. Collection

The wild fruiting bodies of *F. palustris* were collected from a living deciduous broad-leaved tree located in Dalian City, Liaoning Province, China. We took photos of the fresh fruiting bodies in the field. After being dried at 45 °C for 1–2 days, the dried fruiting bodies were stored in the Herbarium of Mycology at Jilin Agricultural University (HMJAU). Referring to the previously described method with slight modifications, briefly, wild fruiting body were washed three times with 75% ethanol, clean tissue inside the fruiting body was cut to 3–4 mm using a scalpel in a sterile bench, transferred to potato dextrose agar (PDA), and incubated for 7 days at 25 °C while protected from light [8]. The pure cultures of the fungus were stored in the Culture Collection at the Mycological Center of Jilin Agricultural University (CCMJ).

#### 2.2.2. Identification

The macroscopic and microscopic morphological identification was carried out according to the Rao et al. Total DNA was extracted from dried fruiting bodies and pure mycelium, respectively, using the new plant genomic DNA extraction kit. The methods of Rao et al. and Wen et al. were used with slight modifications and primers ITS1 and ITS4 were used to conduct the PCR reaction [20,21]. The sequencing work was entrusted to a sequencing company (Sangon, Shanghai, China), and the sequencing results were clipped with Bioedit 7.1.3 and then submitted to GenBank. The sequences of related groups were downloaded from GenBank or obtained from pertinent studies. Maximum likelihood (ML) was used to construct a tree using the IQ-Tree 1.6.8 to illustrate the phylogenetic status of the studied fungus.

### 2.3. Biological Characteristics of F. palustris

The medium was prepared according to different single-factor and orthogonal test requirements in the following proportion [22]: carbon source 20 g/L, nitrogen source 2 g/L, KH_2_PO_4_ 2 g/L, MgSO_4_ 1 g/L, agar powder 20 g/L, 1 mol/L hydrochloric acid, and 1 mol/L sodium hydroxide to adjust pH. The mycelium growth rate (mycelial colony diameter measured in four directions) and growth conditions were recorded every 24 h after inoculation until the mycelium diameter of one of the treatments exceeded 7 cm.

#### 2.3.1. Carbon Source Single-Factor Test

The carbon source candidates included glucose, sucrose, soluble starch, dextrin, and maltose, a culture without adding a carbon source was used as a control. Yeast extract powder was used as the fixed nitrogen source, the pH was not adjusted, and after inoculation, the culture was maintained at a constant temperature of 25 °C.

#### 2.3.2. Nitrogen Source Single-Factor Test

The nitrogen source candidates included yeast extract, yeast extract powder, peptone, sodium nitrite, and (NH_4_)_2_HPO_4_. a culture without adding a nitrogen source was used as a control. Glucose was used as the fixed carbon source, the pH was not adjusted, and after inoculation, the culture was maintained at a constant temperature of 25 °C.

#### 2.3.3. pH Single-Factor Test

Glucose served as the carbon source, and yeast extract powder served as the nitrogen source. Furthermore, the pH values of the medium were adjusted to 5.0, 6.0, 7.0, 8.0, and 9.0 with 1 mol/L HCl and 1 mol/L NaOH, respectively. After inoculation, the culture was maintained at a constant temperature of 25 °C.

#### 2.3.4. Temperature Single-Factor Test

Glucose and yeast extract powder as the carbon and nitrogen sources were used, and the pH was not adjusted. After inoculation, the cultures were placed in incubators at 15 °C, 20 °C, 25 °C, 30 °C, and 35 °C, for constant temperature culture.

#### 2.3.5. Orthogonal Test

According to the results of the single-factor tests, an orthogonal test of four factors and three levels was designed. The medium was made using the corresponding carbon source, nitrogen source, and pH, and the cultures were incubated at the corresponding temperature.

### 2.4. Domesticated Cultivation of F. palustris

#### 2.4.1. Liquid Culture

Liquid culture was performed on the basis of the results of the experiments on biological characteristics in this study. The liquid medium consisted of glucose 20 g/L, yeast extract powder 2 g/L, KH_2_PO_4_ 2 g/L, MgSO_4_ 1 g/L, 1 mol/L hydrochloric acid, and 1 mol/L sodium hydroxide to adjust the pH to 6.0. All media were produced in 250 mL shake flasks containing 120 mL of the culture medium. After inoculation, the flasks were shaken and incubated at 35 °C and 150 rpm/min.

#### 2.4.2. Domesticated Cultivation

The domesticated cultivation was carried out referring to the previous methods of Du et al. and Liang et al. with slight modifications [22,23]. In brief, the raw materials used for cultivation were broadleaf sawdust (poplar) 77%, wheat bran 20%, glucose 1%, lime 1%, and gypsum 1%. The raw materials were stirred, and then water was added to them. Wood chips were kept in this mixture for 12 h to soak up water, and the moisture content was maintained at around 60%. Each cultivation substrate contained 800 g of wet raw material, which was autoclaved at 121 °C for 2 h, and then cooled for 12 h at an ultra-clean bench. Each cultivation substrate included 20 mL of the liquid strain. The culture was then incubated at 35 °C, protected from light, until the mycelium was thoroughly matured in the culture medium. The ambient temperature was lowered to below 25 °C to stimulate primordia formation. A V shape was cut above the primordium after it was formed, the air humidity was increased to about 90%, and the ambient temperature was always maintained at about 35 °C, promoting the maturity of fruiting bodies.

### 2.5. Extraction and Total Sugar Content of Crude Polysaccharides from F. palustris

#### 2.5.1. Extraction

Crude polysaccharides were extracted from the fruiting bodies using the water extraction and alcohol precipitation method. The cultivated fruiting bodies were dried to a constant weight, pulverized, and then filtered through a 40-mesh sieve. Based on the quality of the liquid material, pure water was added at a 1:20 ratio for 3 h in an 80 °C water bath. The aqueous extract was concentrated to one-quarter of its original volume with rotary evaporation. Then, the concentrated solution was added to anhydrous ethanol, giving a final ethanol concentration of 80%, and kept at 4 °C for 12 h. The concentrated solution was then centrifuged at 8000 r/min for 20 min, and the precipitated crude polysaccharides (FPPs) were collected through freezing and drying.

#### 2.5.2. Total Sugar Content

The total sugars in the crude polysaccharides were determined with reference to a previously described phenol–sulfuric acid method with minor modifications [22,24]. A 100 μg/mL standard solution of glucose was prepared as a reference solution. The supplied glucose standard was diluted to concentrations of 0, 10, 20, 30, 40, 60, 80, and 100 μg/mL. Then, 5% phenol and concentrated sulfuric acid were added to the sample and standard glucose solutions. The absorbance was detected with a microplate reader at a wavelength of 490 nm after the reaction was completed. The total sugar content was calculated based on a standard curve.

### 2.6. Determination of Antioxidant Activity of FPPs by Chemical Method

#### 2.6.1. Free Radical Scavenging Assay

Pure water was used to make 0.125, 0.25, 0.5, 1, 2, 3, 4, and 8 mg/mL FPPs solutions and VC solutions. The specific methods and calculations for the hydroxyl radical scavenging assay were conducted in accordance with the instructions provided with the relevant kits.

The DPPH radical scavenging assay references the method of Yarley et al. with some adjustments [25]. In short, a 0.2 mmol/L DPPH solution was prepared using DPPH and anhydrous ethanol as raw materials. A 1 mL volume of the FPPs solution and 1 mL of the DPPH solution were recorded as A1; 1 mL of pure water and 1 mL of the DPPH solution were recorded as A2; 1 mL of the FPPs solution and 1 mL of anhydrous ethanol were recorded as A3. The reaction was performed in dark conditions at room temperature for 30 min, and a microplate reader was used to measure the absorbance at 517 nm. VC was used as a positive control and manipulated in the same way. The scavenging activities were calculated using the formula DPPH·% = [A2 − (A1 − A3)]/A2 × 100%.

#### 2.6.2. Ferric Reducing Antioxidant Power

Pure water was used to make 0.125, 0.25, 0.5, 1, 2, and 3 mg/mL FPPs solutions. The specific methods and calculations for the FRAP were conducted in accordance with the instructions provided with the relevant kits.

### 2.7. Protective Effect of FPPs against Cellular Oxidative Stress

#### 2.7.1. Cytotoxicity Assessment

The cellular antioxidant assay and subsequent determination of antioxidant enzyme activities were performed in reference to a previous method with some modifications [26]. RAW 264.7cells were inoculated into 96-well cell culture plates at a density of 1 × 10^5^ cells/mL, with 100 μL per well. Incubation was performed for 24 h until the cells adhered to the wall, then, the medium was changed to a new medium containing different concentrations of FPPs (0, 50, 100, 150, 200, 250, 300, 350, and 400 μg/mL). After continuing the culture for 24 h, cell viability was assayed using the CCK-8 assay according to the manufacturer’s instructions. 

#### 2.7.2. H_2_O_2_-Induced Oxidative Stress Model

RAW 264.7cells were inoculated into 96-well cell culture plates at a density of 1 × 10^5^ cells/mL, with 100 μL per well. Incubation was performed for 24 h until the cells adhered to the wall, then, the medium was changed to a serum-free medium containing different concentrations of H_2_O_2_ (0, 200, 400, 600, 800, 1000, and 1200 μmol/L). After continuing the culture for 5 h, cell viability was assayed using the CCK-8 assay according to the manufacturer’s instructions.

#### 2.7.3. Protective Effect on H_2_O_2_-Induced Oxidative Stress in RAW264.7cells

RAW 264.7cells were inoculated into 96-well cell culture plates at a density of 1 × 10^5^ cells/mL, with 100 μL per well. The incubation was performed for 24 h until the cells adhered to the wall. Then, the experimental group medium was replaced with a new medium containing different concentrations of FPPs (200, 250, 300, and 350 μg/mL), while the control and model groups were administered with the normal medium. After continuing the cultivation for 24 h, the experimental and model groups were switched to a serum-free medium containing 800 umol/L H_2_O_2_, while the control group was switched to a serum-free medium. After further cultivation for 5 h, according to the manufacturer’s instructions, cell viability was measured using the CCK-8 assay.

#### 2.7.4. Determination of ROS Accumulation

RAW264.7cells (1 × 10^5^ cells/mL, 2 mL/well) were incubated in a 6-well plate. The cells were then divided into groups and treated using the protocol described in Section 2.7.3 to treat them. After the different grouping treatments, the old culture medium was removed and replaced with a serum-free medium containing 10 μM DCFH-DA. The cells were incubated in darkness for 20 min and then washed with PBS. The cells were observed and photographed using a fluorescence microscope, and the mean fluorescence intensity was quantified using ImageJ software (version 1.50i).

#### 2.7.5. Determination of Antioxidant Enzyme Activities and MDA Levels

RAW264.7cells (1 × 10^5^ cells/mL, 2 mL/well) were incubated in a 6-well plate. The cells were then divided into groups and treated using the protocol described in Section 2.7.3 to treat them. The treated cells were collected and lysed on ice. They were centrifuged to collect the cell supernatant, and the SOD, MDA, and CAT levels were measured according to the instructions provided by the kit manufacturer.

### 2.8. Statistical Analysis

All experiments were performed in at least quadruplicate. Data values are presented as mean ± standard deviation (mean ± SD). The results were analyzed by one-way analysis of variance (ANOVA) with SPSS 26.0 and GraphPad Prism 9.5.0. Differences were considered to be statistically significant if *p* < 0.05. The figures were plotted using GraphPad Prism 9.5.0.

## 3. Results

### 3.1. Identification of F. palustris

**Figure 1:**
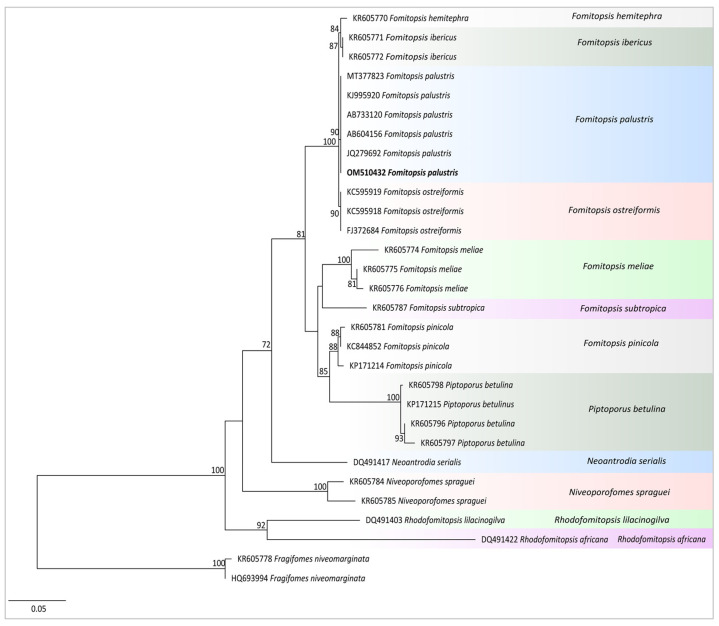
Maximum likelihood phylogenetic tree of *F. palustris* with other closely related species generated from the ITS data set. Newly sequenced collections are indicated in bold.

The morphological characteristics of the fungus are as follows. A distinctive feature of the fruiting bodies is that they are juicy when fresh, relatively soft in texture, and they obviously harden after drying. They are sessile; pileus semicircular; project up to 6 cm, 10 cm wide, and 2–3 cm thick at the center; the pileal surface is light yellowish brown, rough and tuberculate, finely tomentose, and the margin is obtuse. The pore surface yellowish brown, polygonal, or slightly rounded, 3–4 per mm, and the dissepiments are thick. The flesh of the mushroom is slightly lighter in color than that of the cap, and is 1 cm thick. The tubes are concolorous with context, corky, and indistinctly layered. The basidiospores are 6–7.5 × 2–3 µm, ellipsoid, hyaline, slightly thick-walled, and smooth. The macroscopic and microscopic morphology of the wild fruiting body specimens were consistent with the description of *F. palustris* [27]. After sequencing, we found that the sequence of the fruiting body and the mycelium sequence to be consistent, and it was determined that pure mycelium was obtained from the *F. palustris* fruiting body; we then uploaded the fruiting body sequence to NCBI (GenBank accession number: OM510432). Through molecular identification, the sequence was used to construct a phylogenetic tree together with the lines of other related species. According to Figure 1, our sequence clustered with *F. palustris*. Both morphological and molecular systematology confirmed that our specimen was *F. palustris*.

### 3.2. Biological Characteristics of F. palustris

#### 3.2.1. Carbon Source Single-Factor Test

**Figure 2:**
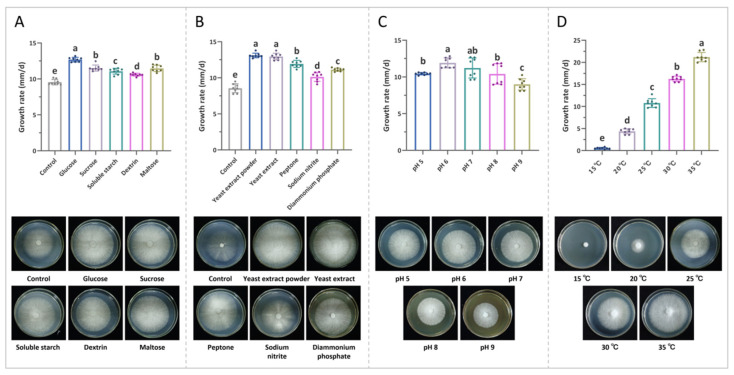
Effects of different single incubation factors on mycelial growth of *F. palustris*. (**A**) Carbon source, (**B**) nitrogen source, (**C**) initial pH, and (**D**) temperature. Different letters (a–e) indicate significant differences among the different samples (*p* < 0.05).

As is evident in Figure 2A, *F. palustris* was able to grow dense white mycelium in a radial pattern using all five carbon sources tested. Glucose was the most rapidly utilized carbon source, followed by sucrose, maltose, soluble starch, and dextrin in descending order of growth rate. Notably, the growth rate of the mycelium in the control group without a carbon source was slightly lower than that in the experimental group, and the mycelium was visibly sparse and weak.

#### 3.2.2. Nitrogen Source Single-Factor Test

The results of the nitrogen source single-factor test can be seen in Figure 2B. Among all the nitrogen sources tested, yeast extract powder was the most effective in promoting *F. palustris* mycelial growth, followed by yeast extract, peptone, diammonium hydrogen phosphate, and sodium nitrite. When organic nitrogen sources were present, the mycelium showed a radial growth of dense and creamy white strong mycelium, whereas with inorganic nitrogen sources, it remained relatively dense or displayed a sparser, yet still white, appearance. Notably, in the absence of a nitrogen source, the mycelium grew slowly and appeared significantly more sparse and weak compared to that of the experimental group.

#### 3.2.3. pH Single-Factor Test

Among the initial pH conditions in this test, the mycelium of *F. palustris* had the best growth performance at pH 6, followed by pH 5, pH 7, pH 8, and pH 9, the detailed results can be seen in Figure 2C.

#### 3.2.4. Temperature Single-Factor Test

As is evident in Figure 2D, among all the temperature conditions in this experiment, the optimum growth temperature of *F. palustris* was 35 °C, at 15–35 °C, the mycelial growth rate increased as the temperature increased. When the ambient temperature was lower than 20 °C, the mycelial growth rate was lower, and when the ambient temperature was higher than 30 °C, the mycelial growth rate was faster.

#### 3.2.5. Orthogonal Test

An orthogonal test of four factors and three levels was conducted, building on the previous single-factor tests (Table 1). The results showed that *F. palustris* could grow dense or comparatively dense and white strong mycelia under each treatment, but the growth rate was somewhat different. The visual analysis displays the range (R) of the temperature factor of 11.67, which was the most important of the four single factors, followed by nitrogen source, pH, and carbon source. According to the mean results, the optimum culture conditions for *F. palustris* were found to be glucose, yeast extract powder, pH 6, and 35 °C, and the results are consistent with the single-factor tests. The analysis of variance (Table 2) showed that the ranking for the F values for the four factors were temperature, nitrogen source, pH, and carbon source. The F values for temperature were significantly higher than those of the other three factors, which is consistent with the visual analysis results.

### 3.3. Domesticated Cultivation of F. palustris

The mycelium exhibited a white color and a prickly texture, and it was evenly distributed throughout the clear golden-yellow liquid medium 4 days after inoculation in the liquid culture. Subsequently, the mycelium pellet matured, and uniformly filled the liquid medium 6 days after inoculation.

**Figure 3:**
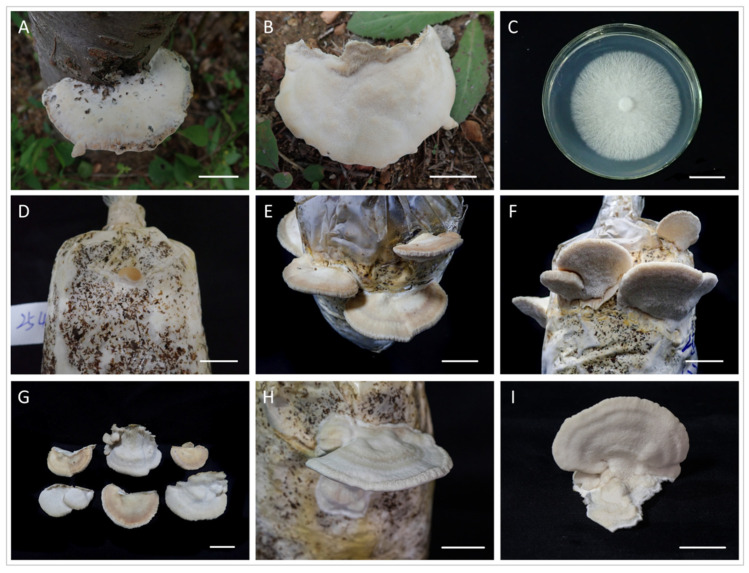
*F. palustris*. (**A**,**B**) Wild fruiting bodies, (**C**) isolated strains, (**D**–**G**) cultivated fruiting bodies at constant temperature (35 °C), and (**H**,**I**) cultivated fruiting bodies at room temperature (17–27 °C). Bars = 2 cm.

The mycelium was grown on the cultivation substrate for 15 days, followed by an additional 7 days of cultivation to complete the ripening process. After nine days of exposure to low temperatures (below 25 °C), light yellowish-brown primordia started to form. And then, with an additional 25 days spent in the greenhouse (around 35 °C), the fruiting bodies finally matured. The morphology of the cultivated fruiting body was highly similar to that of the wild fruiting body (Figure 3D–G). In fact, before the cultivation method described in Section 2.4.2 of this paper, the authors also attempted cultivation at room temperature. However, the mycelium took a longer time to grow on the culture substrate, and the ripening time of the fruiting body was even more prolonged at room temperature in August (17–27 °C). Additionally, the fruiting bodies that matured at room temperature were thinner and had a lighter color (Figure 3H,I).

### 3.4. Extraction and Total Sugar Content of FPPs

The crude polysaccharides extracted from 100 g of dry fruiting bodies amounted to 10.34 g. The standard curve for glucose obtained using the phenol–sulfuric acid method followed the formula Y = 0.0095X − 0.0042 and had a correlation coefficient of R^2^ = 0.9964. The absorbance values measured at the same wavelength for a specific concentration of the FPPs solution were input into the calibration. After calculation, the total sugar content of the FPPs was 26.03%. 

### 3.5. Determination of Antioxidant Activity of FPPs by Chemical Methods

#### 3.5.1. Free Radical Scavenging Assay

**Figure 4:**
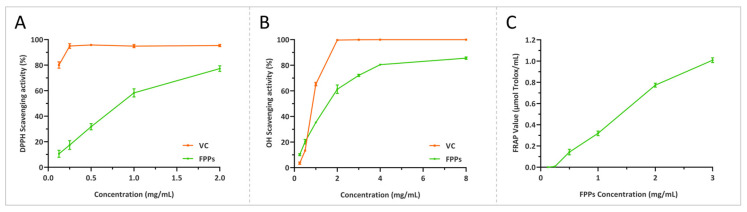
Determination of antioxidant activity of FPPs by chemical method. (**A**) Scavenging activities against DPPH, (**B**) scavenging activities against OH, and (**C**) ferric reducing antioxidant power.

In vitro, free radical scavenging assays are one of the most effective means of assessing the antioxidant capacity of substances [28]. As can be seen in Figure 4A,B, the positive control VC exhibited a strong free radical scavenging capacity. Meanwhile, the FPPs, although slightly inferior to VC, also displayed significant scavenging activity against DPPH and hydroxyl radicals. The scavenging activity against the DPPH radical was found to be 86.52% when exposed to an FPPs concentration of 2 mg/mL, and the EC50 value was 0.81 mg/mL. The scavenging activity against the hydroxyl radical was observed to reach 85.6% with an FPP concentration of 8 mg/mL, and the EC50 value was 1.47 mg/mL. The scavenging rates for the free radicals increased along with an increase in the concentration of FPPs in the concentration ranges tested, which indicated a positive correlation trend. It is worth noting that in this test, due to the presence of ethanol, when the concentration of FPPs exceeded 3 mg/mL, there were varying degrees of precipitation, which might have interfered with the test data to some extent. To ensure the accuracy of the test data, we only used the scavenging activity data within the concentration range of 0.125–2 mg/mL.

#### 3.5.2. Ferric Reducing Antioxidant Power

The FRAP method is based on the reduction of metal ions by the investigated antioxidant and does not specifically focus on free radical scavenging activity. These results can then be used to assess the overall antioxidant activity, with a stronger reducing power indicating a higher level of antioxidant activity. According to the description of the FRAP kit, the standard curve of Trolox’s iron reduction ability is Y = 1.2416X + 0.0134, with an R^2^ = 0.9996, and there is an excellent linear relationship when the absorbance value is below 1.5. Therefore, the absorbance value of the FPPs in the concentration range of 0.125–3 mg/mL was taken, and the amount of Trolox obtained in the standard curve was used to represent the iron reduction capacity of the FPPs. As shown in Figure 4C, similar to the results of scavenging free radicals, the reduced ability of the FPPs to reduce Fe^3+^ increased with an increase in the FFP concentration. At a concentration of 3 mg/mL, the FPPs had the highest reduction ability, and the FRAP value reached 1.02 μmol Trolox/mL.

### 3.6. Protective Effect of FPPs against Cellular Oxidative Stress 

#### 3.6.1. Cytotoxicity Assessment of FPPs

**Figure 5:**
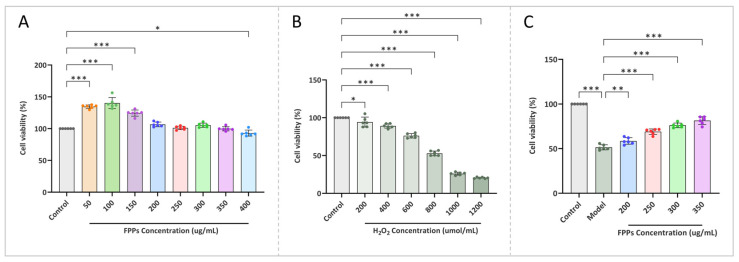
Protective effect of FPPs against cellular oxidative stress. (**A**,**B**) Effect of different concentrations of FPPs and H_2_O_2_ on the viability of RAW264.7cells. (**C**) Effect of FPPs on H_2_O_2_-induced oxidative stress in RAW264.7cells. * *p* < 0.05, ** *p* < 0.01, *** *p* < 0.001.

As shown in Figure 5A, treatment with FPPs at concentrations of 200–350 μg/mL had no significant effect on cell viability compared with the control, whereas treatment with FPPs at concentrations of 50–150 μg/L promoted cell viability to some extent. We finally chose the concentrations of 200–350 μg/mL, which did not have a significant effect on cell viability since the aim of this study was to confirm whether FPPs had a protective effect on H_2_O_2_-induced oxidative cell damage, rather than simply counteracting the damage by promoting cell viability.

#### 3.6.2. H_2_O_2_-Induced Oxidative Stress Model

Currently, cellular oxidative stress models are mainly induced by H_2_O_2_, heavy metal induction, and drug induction, among which, H_2_O_2_ induction is the most common and mature way. It can be observed in Figure 5B that there was a concentration-dependent relationship between H_2_O_2_ and the activity of RAW264.7 cells, and the growth viability of RAW264.7cells gradually decreased with an increase in H_2_O_2_ concentration within 5 h. When the concentration of H_2_O_2_ was 800 µmol/L, the viability of the RAW264.7cells was 52.88%. The concentration of H_2_O_2_ used when the cell viability reaches about 50% is usually used as the model treatment concentration, and at this concentration, RAW264 cells could retain a certain amount of cellular activity while mimicking a state of oxidative stress, so 800 µmol/L was chosen for the subsequent tests.

#### 3.6.3. Effect of FPPs on H_2_O_2_-Induced Oxidative Stress RAW264.7cell

As shown in Figure 5C, the cells in the model group were damaged by H_2_O_2_ and the cell viability decreased to 51.44%. Compared to the model group, the cell viability of the experimental group, which was treated with different concentrations of FPPs, showed a significant improvement and exhibited a concentration dependence. Among these, the 350 μg/mL concentration of FPPs had the most significant protective effect on the cells, increasing the cell viability to 81.36%. This indicates that FPPs could effectively alleviate the oxidative stress caused by H_2_O_2_ on RAW264.7cells.

#### 3.6.4. ROS Accumulation in RAW264.7cells

**Figure 6:**
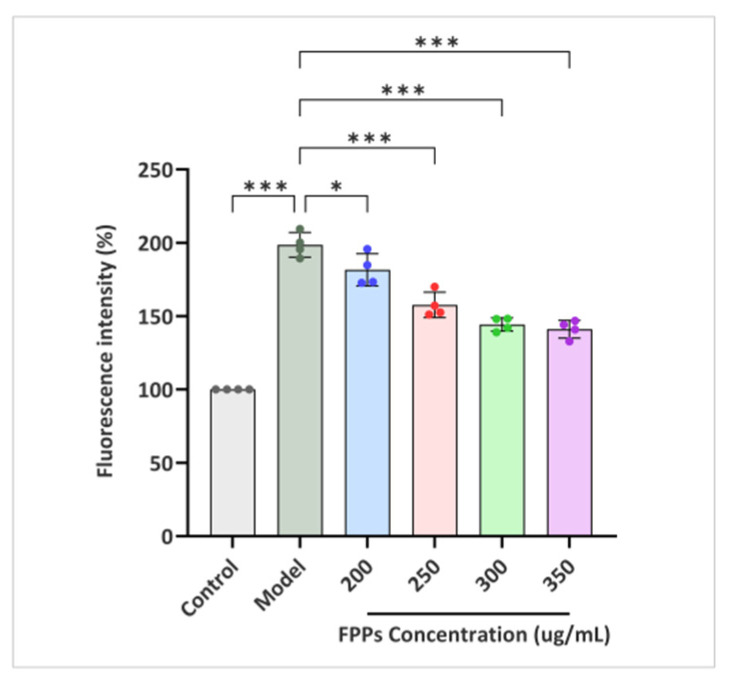
Effects of FPPs on H_2_O_2_-induced intracellular ROS accumulation in RAW264.7cells. * *p* < 0.05, *** *p* < 0.001.

DCFH-DA (2,7-dichlorofluorescin diacetate) is a classical method used for the detection of ROS. DCFH-DA is hydrolyzed by an esterase to form DCFH once it enters the cell. DCFH is oxidized by ROS to form DCF, which emits green fluorescence. Therefore, the accumulation of ROS in cells is indicated by the fluorescence intensity of DCF [29]. Figure 6 shows that the control group had a weaker fluorescence intensity and lower ROS content. Meanwhile, the fluorescence intensity of the model group was significantly increased by H_2_O_2_ induction, which indicated that H_2_O_2_ led to the production of a large amount of ROS in the cells. The fluorescence intensity of the experimental group gradually decreased with an increase in the concentration of the FPPs, and the highest concentration tested, 350 μg/mL, reduced the relative fluorescence intensity to 141.12%. Since we used photography combined with software analysis to quantify the fluorescence intensity of ROS, minor parameter adjustments during the operation may have led to large errors in the results. We tried to keep the parameters as consistent as possible during the operation in order to ensure the stability of the data. However, in order to be able to confirm the antioxidant activity of FPPs more objectively, the subsequent determination of Antioxidant Enzyme (SOD, CAT) Activities, and MDA levels was also essential.

#### 3.6.5. Antioxidant Enzyme Activities and MDA Levels in RAW264.7cells

**Figure 7:**
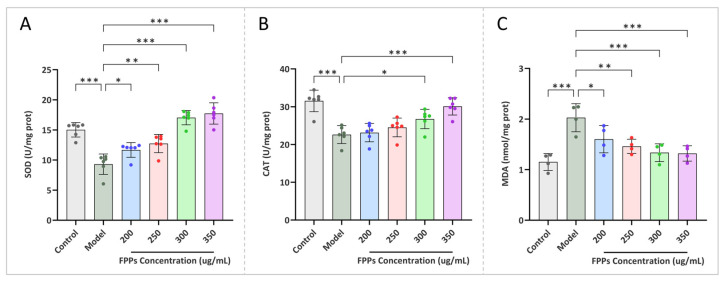
Effects of FPPs on H_2_O_2_-induced intracellular antioxidant systems in RAW264.7cells. (**A**) The activity of SOD, (**B**) the activity of CAT, and (**C**) the level of MDA. * *p* < 0.05, ** *p* < 0.01, *** *p* < 0.001.

Intracellular antioxidant enzymes can play a crucial role in scavenging ROS and preventing cellular oxidative stress. SOD and CAT are important components of these antioxidant enzymes [30]. Figure 7A,B illustrates that, compared with the control group, the model group showed a significant reduction in SOD and CAT activities. This suggests that the intracellular antioxidant enzyme system was disrupted. Compared to the model group, different concentrations of FPPs in the experimental group all improved the unfavorable changes in the relevant indexes in the RAW264.7cells, and there was a concentration-dependent relationship. The optimal concentration of FPPs was 350 μg/mL, which increased the SOD and CAT activities to 18.64 U/mg protein and 29.51 U/mg protein, respectively.

MDA is one of the important end products of lipid peroxidation that can influence the structure and function of the cell membrane by inducing the cross-linking of proteins and nucleic acids, ultimately causing cytotoxic effects [31]. The MDA content is an important parameter that reflects the extent of cellular peroxidative damage [32]. It can be clearly seen in Figure 7C that the MDA content of the model group was significantly higher than that of the control group, indicating that severe lipid peroxidation had occurred in the RAW264.7cells. Compared to the model group, the experimental group showed improvements in lipid peroxidation in the RAW264.7cells with different concentrations of FPPs. This was demonstrated by a gradual decrease in the intracellular MDA content as the concentration of FPPs increased. When the concentration of the FPPs reached 350 μg/mL, it was able to reduce the MDA content to 1.41 nmol/mg protein.

## 4. Discussion

Environmental factors such as carbon source, nitrogen source, pH, and temperature are crucial for mycelial growth, and they are the primary considerations for optimizing the culture conditions. In this study, the optimal culture conditions for *F. palustris* were shown to be glucose and yeast extract powder at pH 6 and 35 °C, through single-factor and orthogonal experiments. Previous related studies have shown that glucose is also an optimal carbon source for the growth of some other mushroom mycelia, such as those of *Auricularia auricula-judae* and *Pleurotus pulmonarius* [33,34]. This may be attributed to the fact that glucose, as a monosaccharide, can be directly metabolized by the mycelium, whereas other relatively complex carbon sources need to be hydrolyzed before they can be utilized. Yeast extract and yeast extract powder are two forms of products derived from the enzymatic hydrolysis of yeast, and there was no significance difference between the two in our experiments, and both are optimal nitrogen sources. Considering that yeast extract is very viscous and not as easy to use as yeast extract powder in our experiments, we chose yeast extract powder as the optimal nitrogen source for the cultures. Clearly, similar to *F. ostreiformis* and *F. pinicola*, the mycelium of *F. palustris* grows significantly better in organic nitrogen sources than in inorganic ones [22,23]. However, unlike them, which show almost no ability to utilize inorganic nitrogen sources, *F. palustris* managed to maintain a relatively good growth rate with inorganic nitrogen sources as well. Interestingly, the test results for pH are different from those of some fungi, which are more suitable for growth in a weakly acidic environment and exhibit poor growth in a weak alkaline environment [22,23]. *F. palustris* can thrive not only in a weakly acidic environment, but it also maintains vitality in a weakly alkaline environment. The existing studies generally agree that 20–30 °C is the optimal temperature range for the growth of most fungal mycelia, for example, the optimal incubation temperature for *Sparassis latifolia* is 24 °C, and the optimal incubation temperature range for *Floccularia luteovirens* is 20–25 °C [35,36]. However, the optimal incubation temperatures of two previously reported fungi of the genus *Fomitopsis* exceeded 30 °C, with an optimal temperature of 31 °C for *F. pinicola* and 35 °C for *F. ostreiformis* [22,23]. This may be a significant difference between fungi of the genus *Fomitopsis* and other fungi, i.e., they are more suitable for cultivation at slightly higher ambient temperatures. The visual analysis and analysis of variance showed that temperature was the most critical factor affecting the mycelial growth of *F. palustris*. This characteristic is of great importance in the liquid culture and cultivation of mushrooms, greatly affecting the productivity of this species. We think that for *F. palustris*, a representative brown-rot fungus, appropriate high temperature helps to promote the enzyme activity of this strain, which in turn promotes the growth of mycelium. When the temperature is too low, even though sufficient nutrients are given, the metabolic requirements for enzyme activity cannot be met and the mycelium cannot maintain good growth.

In the present study, the morphology of the fruiting bodies grown at different ambient temperatures exhibited significant variations. This is consistent with the test results of the biological characteristics in this paper, which also proved that temperature is one of the most critical factors affecting the growth and development of *F. palustris*. A vital characteristic of a potential commercial strain is repaid growth on target substrates intended for cultivation. Although previous reports have briefly mentioned the cultivation of *F. palustris*, after inoculation, it took about 85 days to harvest the fruiting bodies [8]. Our report aimed to provide a more comprehensive and detailed description of the entire cultivation process. After implementing our optimization techniques, which greatly shortened the time required for the liquid cultivation of mycelium and the cultivation of mushrooms, it took about 6 days and 60 days, respectively. The morphology of the cultivated fruiting bodies also exhibited a closer resemblance to that of wild fruiting bodies.

Polysaccharides are one of the main active ingredients of fungi, consisting of one or more monosaccharides polymerized in various linkages to form a long chain of carbohydrate compounds in long chains. Mushroom heteropolysaccharides have a more complex structure than β-glucans, which makes them promising for a wide range of applications in the pharmaceutical and food industries [37]. The total sugar content of the FPPs was 26.03%, which was close to 23.87% of the total sugar content of FOPs, which indicated that the crude polysaccharide obtained contains some impurities such as proteins, and there is still much room for the optimization of the extraction and purification of FPPs [22,38].

Antioxidant activity is one of the most important activities of fungal polysaccharides [28]. In this study, the FPPs showed superior DPPH and hydroxyl radical scavenging activities and ferric reducing antioxidant power. Since the experimental conditions such as reagents and instruments that were used in different studies were not identical, it was not possible to compare the strength of the antioxidant capacity of the FPPs with that of other medicinal fungal polysaccharides in an absolute and objective manner, so we only compared it with that of crude polysaccharides from *F. ostreiformis* (FOPs) obtained under the same conditions. The EC50 values of the DPPH and hydroxyl radical scavenging activities of the FPPs calculated by GraphPad Prism were 0.81 mg/mL and 1.47 mg/mL, respectively, which were lower than those of FOPs (DPPH EC50 value of 1.66 mg/mL and hydroxyl radical EC50 value of 1.68 mg/mL), indicating that *F. palustris* is a medicinal mushroom with a stronger radical scavenging ability [22]. Prasad et al. compared the antioxidant capacity of fruiting bodies and mycelium cultured in liquid medium for 16 different mushroom species, and the results indicated that the fruiting bodies of most species showing slightly stronger antioxidant abilities than the mycelium [39]. In this study, only the activity of *F. palustris* fruiting bodies was investigated, but the advantages of liquid culture in large-scale industrial production are undeniable. Therefore, we believe that subsequent studies comparing the activity of *F. palustris* fruiting bodies with that of mycelium are necessary.

The cellular oxidative stress modeling approach is a classic method used to study the antioxidant capacity of polysaccharides. RAW264.7, as a macrophage line, is often regarded as an ideal cellular model for evaluating the role of bioactive compounds in areas such as inflammation and immunomodulation [40,41]. Notably, there was no obvious promotion or inhibition of cell viability after we added FPPs to the experimental group. However, some of the cells showed more obvious morphological changes, such as an increased size and the growth of pseudopods. Previous studies have shown that certain polysaccharides, such as DRP-B and ASPA80-1, can activate RAW264.7cells from a resting state to an active state, producing morphologic changes similar to those described above that increase the cells’ contact area with external substances and enhance their phagocytosis [16,42]. In addition, there are drugs that are often used to mimic models of inflammation that can similarly activate RAW264.7cells, such as LPS, and these drugs tend to produce more drastic morphological changes, and this overactivation induces inflammation, which can have a range of adverse effects on the body [43]. Fortunately, some polysaccharides such as Rap-1 can modulate this over-activation and thus inhibit the expansion and persistence of inflammation [44]. Clearly, macrophage activation and polarization is a complex life activity, and morphological changes are not the only basis for determining whether RAW264.7cells are activated or not, changes at the molecular level are more noteworthy, such as relevant cell-surface markers [45]. In view of this, we believe that it is necessary to follow up with an in-depth study of the specific mechanisms of FPPs in the areas of inflammation and immunomodulation in the future. Previous studies have shown that some natural polysaccharides have the ability to scavenge free radicals in vitro and can also alleviate oxidative stress in cells by increasing the activity of intracellular antioxidant enzymes and decreasing the levels of ROS and MDA, such as PIP2-1 and MLP-2C [13,46]. Our work demonstrates for the first time that FPPs have a similar antioxidant activity, and furthermore, it was unexpectedly found that the appropriate concentration of FFPs has an effect on macrophage morphology and viability, but the detailed mechanism of action needs to be investigated.

## 5. Conclusions

In this study, a macrofungus was collected from a broad-leaved tree in Dalian, Liaoning Province, and identified as *F. palustris*. The strain exhibited robust growth under the majority of conditions used in this study. The optimal culture conditions included glucose, yeast extract powder, pH 6, and 35 °C. It was observed that temperature had the greatest impact on mycelial growth. After optimization, the time required for the liquid cultivation of mycelium and cultivation of mushrooms was greatly shortened, and the morphology of the cultivated fruiting bodies more closely resembled that of wild fruiting bodies. Furthermore, the raw polysaccharides extracted from cultivated fruiting bodies exhibited potent DPPH radical and hydroxyl radical scavenging abilities, as well as ferric ion reducing capabilities. Even more remarkable is its ability to boost the functioning of intracellular antioxidant enzymes, such as SOD and CAT, to eliminate excessive ROS, lower MDA levels, and effectively mitigate the oxidative stress in RAW264.7cells induced by H_2_O_2_. In conclusion, the ease of cultivating *F. palustris* and its excellent antioxidant capacity amply demonstrate this species’ potential for industrial production and medical research. Our study provides a fundamental basis for the future development and utilization of this species, and we look forward to further research on this species.

## Figures and Tables

**Table 1:** Visual analysis of mycelial growth of *F. palustris*.

Test No.	Carbon Source	Nitrogen Source	pH	Temperature (°C)	Colony Characteristics	Growth Rate (mm/d)
A	1 (Glucose)	1 (Yeast extract)	1 (5)	1 (25)	Comparatively dense, white	9.65 ± 1.10
B	1 (Glucose)	2 (Yeast extract powder)	2 (6)	2 (30)	Dense, white	18.66 ± 0.61
C	1 (Glucose)	3 (Peptone)	3 (7)	3 (35)	Dense, white	20.06 ± 0.41
D	2 (Sucrose)	1 (Yeast extract)	2 (6)	3 (35)	Dense, white	22.11 ± 0.60
E	2 (Sucrose)	2 (Yeast extract powder)	3 (7)	1 (25)	Comparatively dense, white	9.63 ± 0.87
F	2 (Sucrose)	3 (Peptone)	1 (5)	2 (30)	Comparatively dense, white	14.50 ± 0.87
G	3 (maltose)	1 (Yeast extract)	3 (7)	2 (30)	Dense, white	16.18 ± 1.07
H	3 (maltose)	2 (Yeast extract powder)	1 (5)	3 (35)	Dense, white	21.00 ± 0.38
I	3 (maltose)	3 (Peptone)	2 (6)	1 (25)	Comparatively dense, white	8.89 ± 0.52
X1	16.13	15.98	15.05	9.39		
X2	15.42	16.43	16.56	16.45		
X3	15.36	14.49	15.29	21.06		
R	0.77	1.94	1.51	11.67		

**Table 2:** F-test of mycelial growth of *F. palustris*.

Source	Sum of Squares	df	Mean Square	F	Significance
Carbon source	6.58	2	3.29	5.72	0.06
Nitrogen source	37.37	2	18.69	32.47	0.00
pH	23.50	2	11.75	20.42	0.00
Temperature	1242.42	2	621.21	1079.61	0.00
Error	25.89	45	0.58		
Total	1335.75	53			

## Data Availability

Data are contained within the article.
